# Advancements in Nanofiber-Based Electrochemical Biosensors for Diagnostic Applications

**DOI:** 10.3390/bios13040416

**Published:** 2023-03-23

**Authors:** Faiza Jan Iftikhar, Afzal Shah, Qamar Wali, Tayyaba Kokab

**Affiliations:** 1 School of Applied Sciences & Humanities, National University of Technology, Islamabad 44000, Pakistan; 2 Department of Chemistry, Quaid-i-Azam University, Islamabad 45320, Pakistan; 3 State Key Laboratory for Modification of Chemical Fibers and Polymer Materials, College of Materials Science and Engineering, Donghua University, Shanghai 201620, China

**Keywords:** viral diseases, immunology, sensing technology, nanosensors, cardiovascular diseases, diabetes

## Abstract

Biosensors are analytical tools that can be used as simple, real-time, and effective devices in clinical diagnosis, food analysis, and environmental monitoring. Nanoscale functional materials possess unique properties such as a large surface-to-volume ratio, making them useful for biomedical diagnostic purposes. Nanoengineering has resulted in the increased use of nanoscale functional materials in biosensors. Various types of nanostructures i.e., 0D, 1D, 2D, and 3D, have been intensively employed to enhance biosensor selectivity, limit of detection, sensitivity, and speed of response time to display results. In particular, carbon nanotubes and nanofibers have been extensively employed in electrochemical biosensors, which have become an interdisciplinary frontier between material science and viral disease detection. This review provides an overview of the current research activities in nanofiber-based electrochemical biosensors for diagnostic purposes. The clinical applications of these nanobiosensors are also highlighted, along with a discussion of the future directions for these materials in diagnostics. The aim of this review is to stimulate a broader interest in developing nanofiber-based electrochemical biosensors and improving their applications in disease diagnosis. In this review, we summarize some of the most recent advances achieved in point of care (PoC) electrochemical biosensor applications, focusing on new materials and modifiers enabling biorecognition that have led to improved sensitivity, specificity, stability, and response time.

## 1. Introduction

The modern world has seen tremendous advancements in technology, which have resulted in decreased mortality rate, among other benefits; yet, this has occurred with a trade-off: the emergence of new health-related problems [1]. Modern society has ironically been riddled with ever-emerging new strains of viruses that have wreaked havoc on the human population, one of which we are witnessing in our life time is COVID-19, which keeps mutating and striking with more lethality. Producing antibodies against any disease is one of the strongest mechanisms that a body can offer. Antibodies or hormones are released against a disease into the body, which act as biomarkers for accurate, reproducible, and objective measurement of disease by establishing a patient relationship with a clinical end point [2]. The clinical end point may include infection, stroke, cancer, heart disease, HIV, etc., which is supported by epidemiological, physiological, pathological, and therapeutic evidence [3,4].

Contemporary society has been more focused on health and the treatment of diseases. Biosensors have evolved over the years owing to advancements in nanotechnology and the understanding of molecular self-assembly and nanoscale principles. The interaction of a bioreceptor with a bioanalyte results in a transduction signal that is fed to detector for a suitable response to be displayed. Hence, biosensors based on their transduction signals can be classified as electrochemical, piezoelectric, field-effect-transistors, thermometric, and optical detection biosensors [5,6]. Biosensors, especially as electrochemical sensors, have been in demand due to their quick detection and diagnosis of disease using specific biomarkers in the human body. Here, the change in electrical properties of the analyte of interest in terms of ions or electrons is detected as an electrochemical signal due to the electron transfer process, which does not require expensive equipment, as is required for acoustic or optical biosensors [7]. The electrochemical detection method, however, requires high sensitivity and a short range of detection; yet, the detection range has so far been particularly large. This can be improved by modifying the surface of the sensors with molecules such as DNA, enzymes, carbon-based nanomaterials, nanoparticles, and hybrid materials [8,9,10,11]. The oxidative DNA damage caused by pharmaceuticals can be investigated with biomarkers, such as 8-oxoG and 2,8-DHA, that show enhanced signals on glassy carbon electrode when modified by DNA [12]. Similarly, significant work has been carried out by using carbon-based nanomaterials as sensing platform for enhanced efficiency and reaction specificity [13]. Many studies refer to improving the sensing ability by modifying the electrode surface with molecules that lead to an improved limit of detection (LOD) by enhancing the surface area and sensitivity of the sensing matrix for the analyte of interest [14,15,16]. 

Nanomaterials have been found to confer unique properties to sensors by modifying the transduction surface. Nanomaterials typically have at least one dimension that lies between 1 and 100 nm and possess unique physical and chemical characteristics. For instance, due to the small size of nanomaterials, their electrical conductivity and optical properties significantly vary compared with those of their bulk counterparts. These nanomaterials are effective in stabilizing the platform for sensing biological molecules by offering increased surface area, high reactivity, and biocompatibility, as has been discussed in [16,17,18]. Having a high surface area to improve the sensitivity of biosensors along with improved accessibility of the molecule is imperative. One-dimensional nanomaterials such as nanofibers may include synthetic or natural polymers (cellulose), metal-oxide-based nanowires (NWs), carbon nanotubes (CNTs), and carbon nanofibers (CNFs); however, they have not been widely used in the literature for the electrochemical diagnostics of biomarkers. They are promising materials for electrode modification owing to of their structure, which supports efficient electron transport. The conducting features of CNTs are further improved by the incorporation of metal nanoparticles (M NPs). Biocompatible nanomaterials such as iron, gold, zinc, copper, and silver M NPs bestow unique electrochemical recognition capacity to the electrode for the detection of organic analytes. CNTs and CNFs have shown more promise as they offer a long electron pathway and facilitate electrolyte penetration due to their porous nature [19,20], thus improving sensing properties. Nanowires are characterized by a slender structure with excellent electron transport properties, having an aspect ratio (ratio of length to diameter) of 1000 nm, which is smaller than that of nanotubes, which can be used as nanorobots and as microdialysis probe for the detection of glucose in the body [21,22,23]. CNTs have a very high surface area, a higher aspect ratio, and excellent absorption properties. In CNTs, the graphite layer runs parallel to the inner hollow tube; in CNFs, the graphite layer forms an angle with the inner tube axis that can be hollow or solid. This renders the diameter of the CNF to be 10–50 nm; in comparison, CNTs have a diameter of around 100 nm. CNFs have graphite at their edge planes, making the surface highly attractive for the functionalization of materials to form hybrid nanomaterials [24,25]. Electrospinning has been the favored method to synthesize CNFs. It employs the bottom-up approach to form continuous and stable NFs at low cost, while the chemical vapor deposition (CVD) and plasma-enhanced CVD methods have also been adopted as bottom-up approaches. In electrospinning, optimizing the process and solution parameters results in the controlled morphology of the desired NFs [26,27].

In this review, we considered electrospun NF-based nanomaterials, specifically those with a diameter below 1 μm with length spanning several centimeters, and their application to detect biomedical molecules of importance via electrochemical sensing was discussed. This is supported by a conclusion and future prospects on NF-based electrochemical sensors. 

## 2. NFs as Efficient Electrochemical Sensing Platforms

NFs are tiny1D strands of high-surface-area material with unique highly directional strength and flexibility. These NFs have long been under development; yet, they did not gain much popularity until 1990 with the breakthrough advent of nanotechnology. These are synthesized by different techniques employing top-down and bottom-up approaches. The top-down approach utilizes bulk materials to fabricate NFs and includes chemical and mechanical methods such as grinding, milling, etc. The bottom-up approaches, which are widely used to build NFs from basic building blocks, include techniques such as melt blowing, freeze drying, self-assembly, template synthesis, phase separation, chemical vapor deposition, etc. Electrospinning is the commonly used bottom-up technique, which allows control of the NF morphology for achieving maximum performance by controlling the flow rate, concentration of the polymer solution, voltage, distance between the nozzle and collector, etc. Further descriptive details of the techniques to synthesize NFs are described in [28]. A wide variety of polysaccharides, such as alginate and chitosan; and polymers, which can be natural or synthetic, such as collagen, keratin polylactic acid, poly(lactic-co-glycolic acid) (PLGA), composites, and metals/metal oxides, have been used for the synthesis of NFs. Because of their promising and tunable properties, NFs are perfect candidates for biomedical applications. Electrospun NFs are characterized by a high surface area to volume ratio, which allows for more efficient and sensitive detection of analytes. This is because a larger surface area increases the likelihood of analytes binding to the fiber surface, leading to better sensitivity in detection.

Thus, to harness NFs as an efficient platform in biosensing devices and to further optimize the biointerface for enhanced activities, bioreceptor binding event/interactions should be boosted for an accurate, stable, and fast response time, and sensitive analytical performance. To meet this end, special attention has been focused on lab-on-chip and paper-based sensors as point-of-care (PoC) devices, which are used in the healthcare sector for near-patient testing, deployed irrespective of place and time. PoC is an emerging tool for quantifying and diagnosing biomarkers. However, the commercialization of such devices is tedious, and scientists are striving to develop sensors that can be readily used for applications with the minimum expenditure of resources. Here glucometers and pregnancy tests are some of the examples of on-site self-monitoring of health and have been successfully commercialized. 

The electrospinning technique is one of the most commonly used methods to synthesize NFs. The vertical and horizontal set ups in electrospinning result in different electrochemical spun NFs such as single NF and/or NF mats [29,30,31]. These NFs are synthesized by using a range of natural fillers or synthetic polymers, mostly incorporating fillers, nanoparticle (NPs), metal salts, and carbon-based nanomaterials for advanced functionalities. A detailed review on electrospinning can be found in [29,32,33,34]. NFs have a variety of morphologies that are produced by modifying the condition or set up for electrospinning [35]. However, for sensing applications, in order to facilitate the interaction of biological molecules by entrapping them in NF that avoids direct contact with organic solvents, a core–shell NF was devised by using spinneret configuration [36], where injection speed tuned the inner–outer sheath size and thickness. The binding of bioreceptors such as enzymes, DNA strands, antibodies, aptamers, etc. [37,38,39,40,41], to the NFs is carried out by physical or chemical means to induce effective interfacial interaction while retaining the functionalities of hybrid bioreceptor–NF assemblies for detection of analyte of interest. Thus, their superior performance can be attributed to the synergistic role of bioreceptors and NFs.

In order to keep the environment inert for biomolecules, the native structure should be modified in such a way that the reactivity and recognition events are not compromised. Hence, hydrophilic poly(ethylene terephthalate) (PET) and poly(vinyl alcohol) (PVA) have been extensively employed for the purpose; however, to maintain operational stability, they must be further treated by linking to glutaraldehyde (GA), which additionally improves the biocompatibility. Post-treated low-water-soluble NFs can be generated by attaching a hydrophilic polymer/metal oxide, which helps with binding the bioreceptor. Enhanced transduction signals have been achieved by electrospinning NPs/CNTs into nanospun fibers, which improves the electron transfer. For biosensing molecules, NFs have been synthesized by using metal oxides(MO)/carbon/polymers that can be doped with NPs and CNTs for improving conductivity or covered with a conductive layer of polymer [37,42,43,44,45]. 

These bioreceptors are immobilized on the surface of electrode either by absorption [37,46] or covalent bonding when activated with EDC/ NHS [38,47], or Nafion/chitosan/poly (3,4-ethylene dioxythiophene)polystyrene sulfonate (PEDOT:PSS) entrapment [42,43,48], crosslinking with GA [49], or electrostatic interactions [50], which can detect different analytes of interest. CNFs facilitate electron transfer rate due to their conductive nature and support the adsorption of analytes during the preconcentration step of electrochemical sensing. Furthermore, they have different specificities for different organic analytes, thereby improving the discrimination between the target species on the basis of potential. Moreover, the functional groups of CNFs can hydrogen bond with the analytes’ functional groups (H/O–H) and with the electrode-surface-adsorbed analytes and their aqueous-dissolved species. The rings of CNFs can offer additional π–π interactions to the benzene unit of targeted organic species via solute–sorbent interactions to boost their localized concentration and enhance electrocatalytic redox events. The combined effect of all the above-mentioned interactions gives rise to a sensitive electrochemical scaffold for the discrimination and concurrent biosensing of all the multiple analytes formed due to biochemical reactions in the body such as glucose, uric acid, catechol, H_2_O_2_, etc.

**Figure 1:**
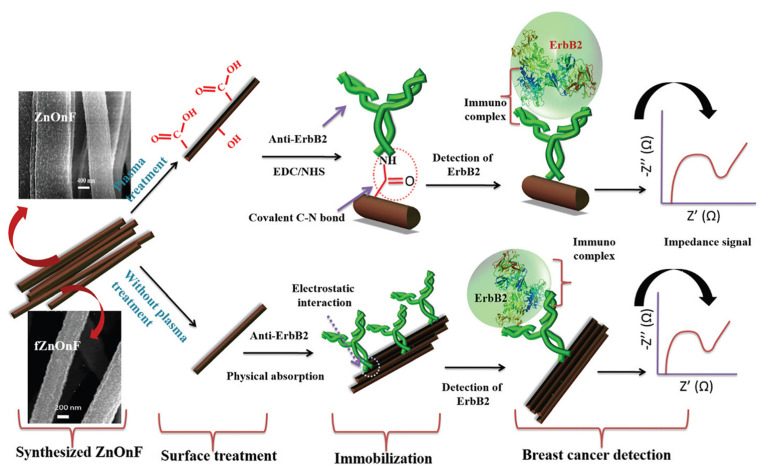
Development of label-free immunosensor for detection of ErbB2 as biomarker for breast cancer by plasma treatment of ZnO NF. Reprinted with permission from [55]. Copyright (2023), RSC.

MO-based NFs have been synthesized using sacrificial polymers as carriers such as PVP with metal salt, which is then calcined at high temperature to remove PVP while the precursor is oxidized to nanocrystals [51,52]. MOs, by a process called nucleation, grow and align along the direction of the electrospun NFs. The as-obtained NFs have a large surface area with improved optoelectronic properties. Here, the metal/metal oxide NPs exert their electrocatalytic role for biosensing via metal nuclei active sites, and the mechanism follows the conventional heterogeneous catalysis process. Calcination and the concomitant loss of polymer result in the shrinkage in the NF diameter, which causes it to be brittle. The resulting mechanical stress also affects the stability of the sensors, which is improved by blending additives during electrospinning. The process is more complex than producing pristine polymer NFs. Mercapto propyl phosphoric acid (MCPPA)/Cu-doped ZnO NFs prepared from PVA and zinc acetate were immobilized on a glassy carbon electrode (GCE) for the detection of antigen histidine-rich protein 2 (HRP2) for the early and quick detection of malaria with an LOD of 0.6 attogram/mL with high specificity [53]. The HRP2 antigen is released by the lethal malarial parasite into the blood stream of the body. Previous work focused on growing CNFs on glass microballoons with HRP2 as an immunosensor for the detection of a specific antibody via calorimetric or electrical signals [54]. Similarly, Cu-doped ZnO NFs were developed for impedimetrically targeting the same analytes as in [53,54]. Cu was found to improve the sensitivity of the sensor by overcoming the intrinsic resistivity of the ZnO NFs. This work could be expanded to include other biomarkers of importance. By introducing NFs onto electrodes, the scalability of NF processing is likely, but comes at the cost of developing it in relation to a specific antigen for the detection of malaria. MCPPA doping helps with immobilizing the anti-HRP2 antibodies by introducing the functional groups such as –COO^−^, etc. The porosity of the sensitive layer offers high-mobility paths and surface activities for target molecules, promoting their interaction and mass transportation across the sensing surface. Cu doping generates an electric field at the interface of the Cu/ZnO heterojunction, along with improved conductivity of NFs. Nano-based immunosensors have been proposed to be used in PoC diagnostic kits. Similarly, a label-free immunosensor for the detection of the breast cancer biomarker, epidermal growth factor receptor 2 (ErbB2), was reported. The PoC device was constructed by using carbon-doped mesoporous ZnO NFs via electrospinning, with fibers ranging in diameter from 50 to 150 nm. The conjugation of ErbB2 to the sensing platform was improved by oxygen plasma treatment, which introduced functional groups such as –COO^−^ and –OH^−^, leading to a high sensitivity of 7.76 k Ω μM^−1^ detected with EIS, as shown in Figure 1 [55]. 

MWCNTs imbedded into crystalline ZnO NFs are covalently bonded to the antibody for the detection of carcinoma antigen-125, which is an ovarian cancer biomarker and can be used for PoC diagnostics due to its high sensitivity and rapid detection. Calcination is optimized to prevent the decomposition of CNTs, and the introduction of –COO^−^ groups helps to bind the antibody to the sensing matrix via the amino groups on the specific antigen via the EDC/NHS coupling reaction. These oxygen functionalities of the *f*CNTs act as anchoring sites to form hydrogen bonds, which are responsible for the increased adsorption of target analytes [56]. TiO_2_ NFs have been used to detect malignant cells from patient suffering from colorectal or gastric cancer [57]. Label-free biosensors can detect biomarkers without the need for labeling agents, which can simplify the detection process and reduce the cost of biosensors. Researchers are exploring the use of nanofiber-based biosensors for the label-free detection of biomarkers. An immunosensor based on the wire-in-tube architecture of chitosan modified IrOx NF, where (0 ≤ x ≤ 2) was synthesized by electrospinning for the label-free detection of the α-fetoprotein (AFP) cancer biomarker by controlling the annealing temperature, resulting in a unique morphology with improved surface area. Thus, it was observed that independent nanowires were incorporated into the NF that were separated along the whole length of the NFs. The antibody (Ab) adsorbed on to the highly oriented modified NFs for the amperiometric detection of AFP in human serum with an LOD of 20 pg/mL was confirmed [58]. 

## 3. Electrochemical Biosensors Based on One Dimensional Electrospun NFs

### 3.1. Detection of Biomarkers for Immunological Diseases

Type 1 diabetes is an autoimmune disease that occurs due to the malfunctioning of the normal healthy tissues that make insulin and disturb its balance by producing more glucose in the body. Electrochemical monitoring sensors for glucose are employed for the fast and sensitive detection of glucose, which can either be enzyme- or non-enzyme-based sensors [59]. Glucose oxidase, GOx, is an enzyme that catalyzes the oxidation of d-glucose to H_2_O_2_ and gluconic acid, which is the technique widely used in glucose biosensors. These sensors are developed either by enzyme immobilization in a suitable matrix by covalent attachment, physical adsorption, or encapsulation, or non-enzyme-based sensors [60].

#### 3.1.1. Metal-Based NFs Immobilized with Enzyme for Detection of Glucose

ZnO NF immobilized on Au electrode coated with a PVA film was employed for the amperiometric detection of glucose [37]. This assembly was further decorated with GOx via physical adsorption and demonstrated an LOD of 1 μM in a linear range of 0.25–19 mM with a very fast response. Similarly, TiO_2_ NF mats immobilized on a Pt electrode and drop-coated with GOx supported by chitosan film were developed [38]. TiO_2_ NFs were found to increase the response of the electrode toward glucose detection by a factor of 2.7 compared with that of pristine Chit/GOx/TiO_2_ film/Pt. Chitosan was added to improve the immobilization of GOx with enhanced sensing as a result of the TiO_2_ NF architecture. However, it was contended that the optimal concentration of TiO_2_ NF is needed for sufficient adsorption of GOx for improved electron transduction. GOx-modified Mn_2_O_3_–Ag NF was used for the detection of glucose, where the PVP acted as a sacrificial polymer, and AgNO_3_ as added to the electrospinning solution to form highly porous NFs. The glucose was amperiometrically detected with this assembly, with an LOD of 1.73 μM [42]. Here, Mn_2_O_3_ offered an interface for the immobilization of the biomolecule, while Ag NP coalesced with it to form a highly porous structure of Mn_2_O_3_-Ag NF. This led to the high loading capacity of the GOx and resulted in a promising sensing matrix. A CNF-modified bimetallic CuCo alloy nanocomposite, to improve the electrocatalytic activity, was synthesized by electrospinning for the detection of glucose in human urine with high sensitivity in a linear range of 0.02–11 mM [61].

#### 3.1.2. Metal NFs for Nonenzymatic Detection of Glucose

**Figure 2:**
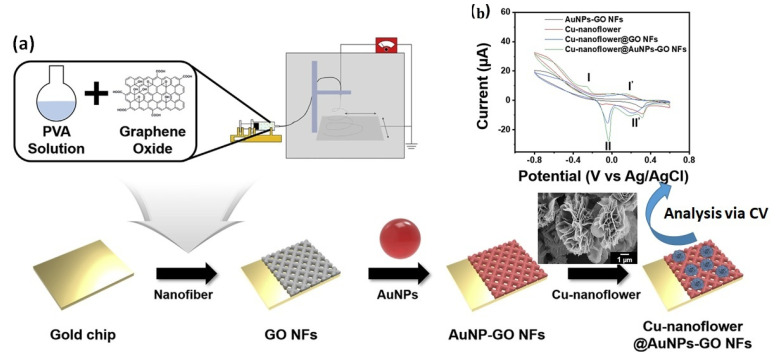
(**a**) Construction of Cu-nanoflower-based AuNPs/GO-modified NFs; (**b**) modified NF employed for detection of 30 μM glucose via CV at a scan rate of 20 mV/s, showing high current response for modified electrode. Adapted with permission from [62]. Copyright (2023) Elsevier B.V.

Metal and metal–oxide composites demonstrate promising signal amplification and electrocatalytic properties for nonenzymatic glucose sensors. This was specifically investigated due to the stability and repeatability issues in enzyme-based nanobiosensors. The embodiment of Cu nanoflowers onto AuNPs via layer-by-layer deposition onto electrospun graphene oxide (GO) from PVA was carried out to form a composite NF for the detection of glucose with improved sensitivity [62]. Cu nanoflowers are expected to improve the surface area of the NFs and, hence, their enzyme activity. The construction of a glucose biosensor is shown in Figure 2. Glucose was successfully detected in the saliva by incorporating Cu NPs onto polycaprolactone@polypyrrole NF on an ITO electrode, which resulted in a synergistic effect to enhance the performance of the sensor. The core–shell NF structure is responsible for enhancing the surface area to improve sensitivity, while CuO NPs improve the conductivity of the sensing matrix, resulting in the oxidation of glucose via the Cu redox couple at the interface [63].

Noble metals such as AgNPs were electrospun with Cu(NO_3_)_2_ and PVP to form AgNP/CuO NF at an ITO electrode with enhanced analytical performance, which retained 93% of the response even after one month of storage [64]. The incorporation of graphene nanomaterials along with transition metals (TMs) boosts the electrocatalytic property of the oxidation of glucose along with improved conductivity of the nanocomposite. The TMs most popular for their electrocatalytic ability, such Ni and Co, otherwise show poor conductivity [65]. Along this line, FeCo CNF was synthesized by electrospinning, followed by pyrolysis of the polymer, which was later dispersed in Nafion. This resulted in a 3D-alloy-based nanosensor with mesoporous channels for the enhanced mass transportation and electro-oxidation of glucose [66].

Similarly, ZnO NFs have a high surface area and a sensing response owing to their features that support electron communication within the structure. This is due to their tiny grain size, which improves their electrocatalytic ability [37]. Electrospinning is employed to form ZnO/PVP composite NF from the respective precursors, followed by calcination at high temperature, resulting in ZnO nanoparticle formation, which is then embedded onto a Au electrode for the detection of H_2_O_2_ formed due to the oxidation of GOx, which is reduced to GOx (R) in the presence of oxygen [67].

#### 3.1.3. Carbon-Based NFs for Detection of Glucose

##### Enzyme-Based Carbon NFs

Carbon is one of the most commonly used materials for sensor applications, such as in such CNTs, which are integrated into the biosensor for achieving high sensitivity. NFs, due to their high surface area and access to functional groups, can load larger biomolecules with higher sensitivity than CNT-based biosensors. The introduction of carbonaceous-nanomaterials-based NFs (CNFs) is the most efficient approach to improve the sensing response, selectivity, and response time. These CNFs are fabricated by electrospinning methods that improve the morphology of electrospun fibers and their surface area. Here, PAN has been found to generate superior properties for electrospun NFs, which include high carbon yield and mechanical strength, and are synthesized with a two-step process [68,69]. This includes the conversion of thermoplastic carbonaceous NFs to thermoset by oxidation at low temperature in an inert atmosphere, which is followed by carbonization at higher temperatures. The surface chemistry and the microstructure of the NFs can be tuned by changing the electrospun solution composition or by postmodification methods such as post heat treatment, which results in the orientation of carbon NFs and improved crystallinity. Moreover, the high-temperature treatment of poly(methyl methacrylate) (PMMA) electrospun onto a Si substrate modified with PAN/chloroplatinate results in Pt-NPs-modified microchannels in a monolithic amorphous carbon electrode. Plasma treatment introduces carboxylic acid groups to allow facile grafting of antiaflatoxin B1 (AFB1) antibody using EDC/NHS at the electrode, where the microchannels act as nanorectors for antibody–antigen interaction. This results in improved charge transport in the channels. The sensing platform is able to detect AFB1 antigen with an LOD as low as 1 pg/mL [70]. In another attempt, silica NPs were synthesized by electrospinning by employing a PAN precursor solution. In order to improve porosity, thermal treatment was carried out that led to orientation effect in carbon fibers that allowed for improved immobilization of GOx. Subsequently, the resulting CNFs were carbonized. The mesoporous carbon thus fabricated was immobilized with GOx, which was easily accessible to glucose molecules [71]. GOx immobilized on to electrospun PMMA entwined with a cationic polymer poly(diallyldimethylammonium chloride) PDDA was dispersed in MWCNTs, which was successful in sensing glucose with an LOD of 1 μM at the modified ITO [72]. PMMA helped to prevent the aggregation of MWCNTs along with the attachment of negatively charged GOx to the modified ITO. This resulted in the pronounced electrocatalytic activity of the modified electrode toward the oxidation of glucose. The conductive polymer PEDOT has been most commonly used for the immobilization of enzymes [73]. 

**Figure 3:**
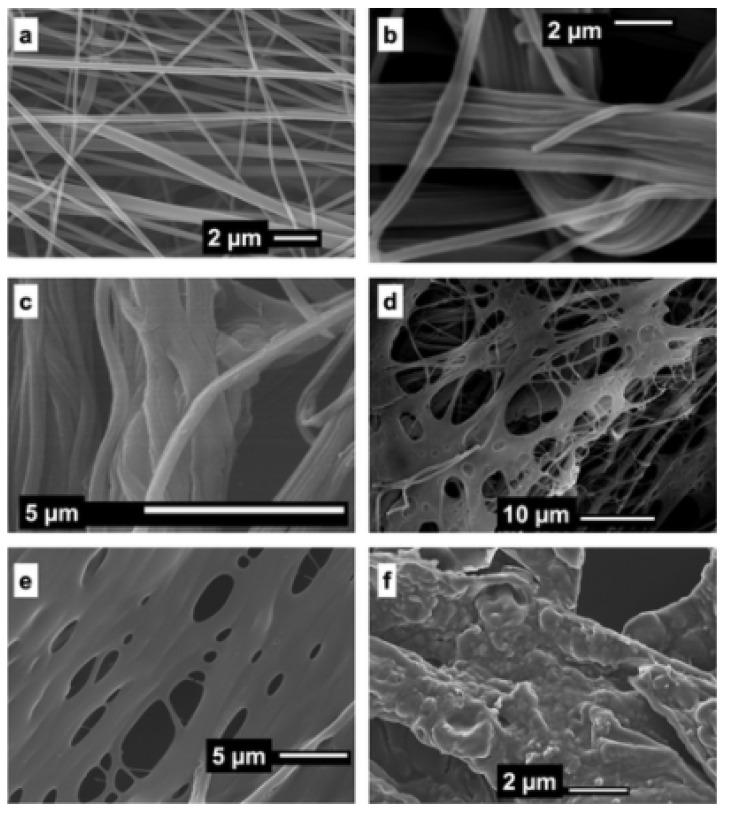
SEM images of (**a**) typical electrospun PAN/PAA nanofibers and (**b**–**f**) graphene wrapping process that shows (**d**) agglomeration as sheets of the graphene wrap instead of enfolding individual fibers, which indicates high-fiber-density areas. (**e**) Smooth NF after carbonization. (**f**) Carbonized graphene-wrapped NF showing the texture of the surface. Reprinted with permission from [76]. Copyright (2023), American Chemical Society.

Similarly, modified poly(L–lactide) (PLLA) NFs have been employed for the detection of glucose. PLLA NFs were immobilized with GOx-PEDOT by the electropolymerization of PEDOT and GOx together, which prevented the leaching of enzyme. This resulted in improved sensitivity compared with that of a PEDOT/GOx matrix on a Pt electrode [43]. Enzymes such GOx can be entrapped in NFs by electrospinning a blend of PVA and GOx, which is crosslinked by using a GA vapor that forms water-insoluble NFs. These NFs have more enzyme loading and better electrode sensitivity than an enzyme attached to NF after electrospinning [74]. The conductivity is further enhanced when PVA and carbon NMs such as GO are incorporated by electrospinning along with chitosan, GO, and GOx to form NFs with high sensitivity. This sensing matrix was able to detect glucose in human serum [75]. Recently, an effective method to develop PAN/PAA nanospun fibers wrapped with graphene (see Figure 3) was reported, which improved the conductivity and active surface area by many orders of magnitude compared with that of carbon-screen-printed electrodes and has the potential to be used as a sensing matrix for bioactive molecules [76].

**Figure 4:**
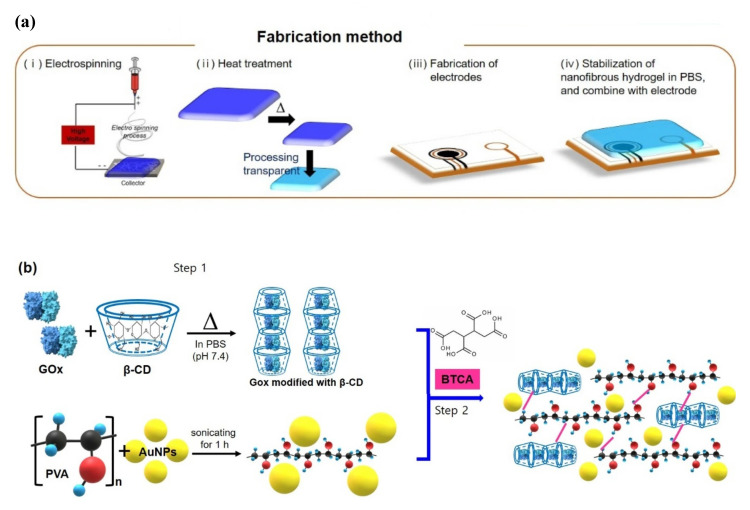
(**a**) Schematic illustration of sensing platform as PVA/BTCA/β-CD/GOx/AuNPs NF hydrogels for glucose detection on SPE; (**b**) preparation of PVA/BTCA/β-CD/GOx/AuNPs by electrospinning. Reprinted with permission from [81]. Copyright (2023) Springer Nature Limited.

Cyclodextrin and cellulose were fabricated as electrospun cellulose/β–CD NFs and employed as a wearable biosensor of glucose via the skin, interlocking the hydrophobic groups of GOx with the hydrophobic core of β–CD, which stabilizes the GOx within the NF. This conferred a high surface area to the NF for glucose monitoring [77]. The sensors without the need for mediators are the fourth-generation sensors, which are preferred to prompt direct electron transfer (DET) reactions. This allows the shuttle of electrons between the active enzyme and the electrode; yet, such sensors are challenged by thermal stability and cost, along with the tedious immobilization of enzymes, which makes these enzymes inactive on the surface of the electrode, rendering the redox centers in the enzyme molecule inaccessible [78,79]. Thus, the inclusion of a mediator for DET is unavoidable. Carbon-based NFs modified with nitrogen-doped carbon nanospheres were prepared by electrospinning polypyrrole (PPY) in a controlled N_2_ environment with adsorption of GOx on GCE, which was further coated with Nafion to retain the stability of the electrode. A NCNSs@CNFs matrix served as a stable platform to favor the DET of GOx without its pretreatment. Nitrogen doping confers hydrophilicity and, hence, biocompatibility, along with improved surface area. This results in a sensitive and stable glucose sensor with an LOD of 2 mM as a result of the direct transfer of electrons between the GOx and GCE. Doping significantly improved the electron transfer compared with that of a CNF/GCE matrix [80]. A novel hydrogel NF material with improved pore size and high surface-to-volume ratio was synthesized by crosslinking 1,2,3,4-butanetetracarboxylic acid (BTCA) with the OH groups of PVA and GOx-encapsulated β–CD mixed with AuNP by electrospinning. PVA/BTCA/β-CD/GOx/AuNPs hydrogel NFs showed improved sensitivity for glucose detection in the interstitial fluids of the body and is a promising candidate for wearable sensors (refer to Figure 4) [81].

##### Non-Enzyme-Based Carbon NFs

Nonenzymatic sensors are also faced with challenges such as a low rate of glucose oxidation as well as oxidation of interferents in the working range, along with adsorbing intermediates as a result of irreversible oxidation, poisoning of the noble metal, and exhibiting low selectivity. Here, noncatalytic material for sensing applications can be employed to overcome these challenges for the nonenzymatic detection of glucose with high sensitivity and selectivity. Carbon nanofibers (CNFs) have a large surface area and facilitate electron transport with improved conductivity in one direction [82]. Mei et al. fabricated Ni/CoOmodified carbon nanofibers by an electrospinning to detect glucose in the body by increasing the electrocatalytic properties of the matrix [83]. The basic idea of increase in the sensing response depends on the formation of a heterojunction between carbon materials and MO because they have different work functions and semiconducting properties. Long-term tissue damage by implantable electronic sensors due to mechanical mismatch, as well as instability in in vivo environments, can be overcome by employing hierarchical fibers that mimic the native tissue in the body with a bending stiffness of less than 10^−3^ nNm^2^, as well as provide mechanical strength [84]. Thus, modified MWCNTs that were twisted to form hierarchical helical fibers to mimic the soft tissue structure such as muscle have been used for in vivo monitoring of biomarkers such as H_2_O_2_ in mice and venous blood glucose in cats via redox reactions, whereas calcium ions and prostate-specific antigens (PSAs) have been investigated via impedance changes with high specificity and reproducibility. These nanofibers can be fabricated as single-ply sensing fiber SSF or multiple-ply sensing fiber MSF for the detection of specific or multiple biomarkers. The SSF for H_2_O_2_ detection can be easily carried out by modifying CNT fiber with Pt, showing a sensitivity of 0.84 μA μM^−1^. For PSA, the SSF is developed by modifying CNT fiber with GO functionalized with chitosan-Pb_2_[Fe(CN)_6_]-PDDA. The detection limit was <0.5 ng ml^−1^ in blood. These sensing fibers are conveniently implanted via a syringe and delivered to the target area, such as to a tumor, by a minimally invasive procedure compared with surgery. Ca^2+^ ion SSF sensors have the same structure as PSF SSF prepared by dip-coating CNT fiber in PEDOT:PSS solution, which is determined by the change in the electric field at the interface of electrode. For enzyme GOx-based glucose-sensing SSFs, a layer-by-layer structure was designed by coating CNT fibers with chitosan, further improving the micropore structure, which was additionally coated with Nafion and GA, resulting in improved stability and selectivity of the SSF. The sensor demonstrated a sensitivity of 5.6 nA μM^−1^ in a working range of 2.5–7.0 mM, exceeding that of a standard glucometer. These NFs have a porous structure that provides a larger space for biomarkers to interact with the fiber surface. This increases the probability of analyte–fiber interactions, leading to enhanced detection sensitivity. Nonenzymatic glucose SSFs were designed by using ZnO NPs–modified CNT nanofibers. For real-time multiplex monitoring, MSF was fabricated by twisting several SSFs together that can detect different analytes in mixed solutions. The high sensitivity of the sensor is related to the application of potential, which helps promote electrons from glucose molecules to the ZnO NPs surface, due to the presence of intrinsic oxygen vacancies coupled with the excellent conductivity of CNTs, which facilitates their electronic mobility. 

### 3.2. Detection of Biomarkers for Cancer

Cancer is one of the most lethal diseases that affects almost 10 million people worldwide [48]. Because biomarkers are very low in concentration in the early stage of disease, sensitive methods are required for early stage detection to save time and life by measuring the relevant cancer biomarkers. 

**Figure 5:**
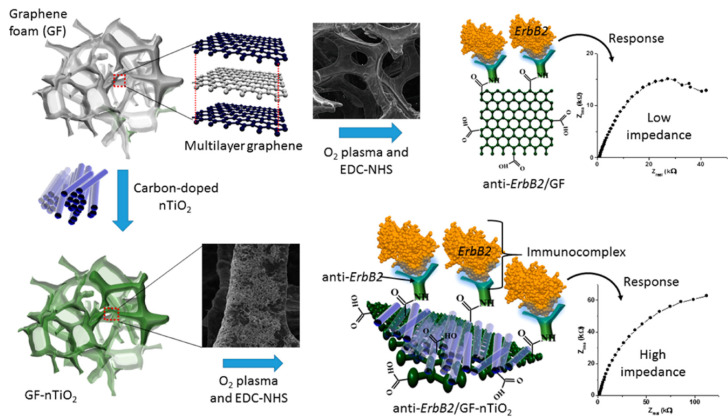
Functionalization of GF and GF–nTiO_2_ NF modified electrodes with the respective SEM images for detection of ErbB2, where the immunocomplex is confirmed via Nyquist plots for the modified electrodes employed in microfluidic devices. Here, GF shows the least impedance. Adapted with permission from [91]. Copyright (2023) American Chemical Society.

Breast cancer is the fifth leading cause of mortality in the world [85]. The most common diagnostic biomarkers for breast cancer are mucin-I and CEA. Although mucin-I is overexpressed in all cytoplasmic and periplasmic cancer of breast [86], it does not help in the early detection of cancer. CEA is of more significant value. A cytosensor, MCF-7, was developed for simultaneously detecting CEA and mucin-I on the surface of breast cancer cells, but it demonstrates a low LoD. Detecting CEA with mucin-I could provide more accurate results and maximize the efficacy and planning of treatment depending on the type of cancer cell [87]. Similarly, another crucial breast cancer biomarker, human epidermal growth factor receptor 2 (HER-2), was detected in serum samples by developing a low-cost and effective immunosensor based on AuNS modified on a laser-scribed graphene (LSG) electrode, demonstrating an LoD of 0.008 ng/mL and good reproducibility. The LSG, due to the formation of defect-free stacked graphene, had a high surface area and offered high electrocatalytic activity with 3D porosity. The new electrochemical sensor exhibited enhanced sensitivity compared with that of bare LSG and commercially available SPAuE. This was tested as a PoC diagnostic device by using a custom-made mobile application [88]. An efficient, label-free, selective, and highly reproducible immunosensor with unprecedented sensitivity (femto-molar) was developed to detect a breast cancer biomarker for early diagnostics. Electrospinning was used to synthesize mesoporous ZnO NFs with a diameter ranging from 50 to 150 nm for the label-free detection of breast cancer. The carbon-doped ZnO NFs, after covalently or electrostatically binding with the anti-ErbB2 biomarker, was deposited onto an ITO glass substrate. The covalent bonding of anti-ErbB2 with the NF was facilitated by oxygen plasma treatment, which introduced functional groups for the effective conjugation of the biomolecule with the electrode. Carbon doping provides high electrical conductivity, biocompatibility, and chemical and thermal stability. Furthermore, the functionalization of NFs via organic or inorganic moieties significantly improves their dispersion and compatibility with biological molecules. Their surface reactivity and active sites render them efficient for enhanced electrochemical responses. The sensing surface possesses electrode-anchoring hydrophobic parts and analyte-interacting functionalities, acting as a connecting bridge and facilitator of electron transfer between the sensor (host) and guest (target molecules). The detection is carried out by an EIS that exhibits an LoD of 7.76 kΩ μM^−1^ and a detection of 1 fM, i.e., 4.34 × 10^−5^ ng mL^−1^, which is a result three orders of magnitude better than that of ELISA. Overall, the nanocomposite acts as a highly active platform because of the synergistic effects of its components. This proposed PoC is expected to be useful for the detection of other bio– and cancer markers [55]. For the efficient sensing of biomarkers, integrating NFs into the microchannels on lab-on-a-chip is quite a difficult task. Researchers [89,90] have improved technologies to form thin-layered NF mats on PVA substrates, which are then incorporated into microchannel as thin strips for the chemiluminescence detection of *E. coli*. In the same fashion, a label-free immunosensor biochip for the detection of breast cancer biomarker ErbB2 was obtained by fabricating NFs employing electrospun carbon-doped TiO_2_ on hierarchical porous graphene foam, as shown in Figure 5. The antibody was covalently immobilized at the surface of a modified electrode via covalent bonds, which resulted in high specificity and selectivity. The TiO_2_ surface, due to the presence of intrinsic oxygen vacancies coupled with the excellent conductivity of carbon and graphene network, facilitated the electronic mobility of the antibody [91]. 

A MWCNT-functionalized carbon paste electrode with an electrospun polyethersulfone ribbon and a conductive nanofiber-based capture probe for the early detection of BRCA1 gene sequence (DNA–t) was developed by inducing the hybridization of DNA–p with DNA–t, which signify probe and target DNA, respectively. The EIS detection limit was determined to be 2.4 pM, with high reproducibility and sensitivity [92]. The chemical functionalities of the probe can act as anchoring sites that interact with the O–H/N–H/O–C groups of the BRCA1 gene sequence (DNA–t), forming hydrogen bonds that lead to their increased adsorption. Additionally, the conjugated ring structure of CNTs offers π–π interaction with the analyte aromatic units, together with specific solute–sorbent interactions, which play a role in their recognition. Moreover, bimetallic Au-Ag/Co_3_O_4_ NFs, synthesized by electrospinning followed by calcination, were used as a sensing matrix for the electrochemical detection of H_2_O_2_ released from breast cancer cells [93]. These biocompatible nanomaterials Au-Ag/Co_3_O_4_ bestow unique electrochemical recognition capacity on the sensing matrix. These noble bimetallic alloy nanoparticles, in conjunction with NF, demonstrate exceptional affinity for analytes of interest compared with their single-metal analogues. Here, the synergistic effect of Au and Ag results in an enhanced surface-to-volume ratio of the as-synthesized NFs, resulting in boosted sensitivity and an LoD of 1241.1 μA mM^−1^ cm^−2^.

Lung cancer is the leading cause of mortality in men, with a high incidence among them, and is the third leading cause of mortality in woman after breast and colorectal cancer. Neuron-specific enolase (NSE) is an important biomarker of small-cell lung cancer, used for its diagnosis and treatment [94]. The increased interest in label-free immunosensors is due to the fact that they are easy to construct and elicit fast response. Conducting polymers such as polypyrrole (PPY), due to their biocompatible nature and high stability, have been extensively deployed for biosensor design. These biosensors are additionally integrated with reduced GO, Au NPs, and other polymers as nanocomposites to improve the sensitivity of the biosensor. Epoxy-functionalized PPY has epoxy side-groups that are important for binding the amino group of the biomolecule (anti-NSE antibodies) and improving the surface area. Its synthesis is cost-effective, offering a simple method for biosensing. Tin oxide modified with epoxy-functionalized PPY on ITO was reported as a label-free immunosensor for detection of NSE, where the epoxy-functionalized PPY was employed for the first time [95]. 

Melanoma, an aggressive skin cancer, has high rate of survival when localized in skin; however, the survival rate precipitously drops when it metastasizes first to the lymph nodes and adjacent organs and then subsequently to distant organs [96,97]. Many tissue-based biomarkers are important indicators of the primary lesion; however, none give a satisfactory count of how many cancer cells have entered into blood stream as a result of dislodging from the lesion and becoming a seed for metastasis, which are known as circulating tumor cells (CTCs). To overcome the limitations of the existing diagnostic tools [98,99,100], a highly selective and sensitive method for its detection needs to be developed, which is easy to operate and gives a rapid response [101,102]. For these PANI NFs, which are noncytotoxic and biocompatible, were employed by loading antibody via covalent bonding, which is highly desirable as a biosensor design. Hence, PANI NFs functionalized with anti-MC1R antibody on SPE were reported as selective and sensitive immunosensors to detect melanoma cells (SKMEL-2) with a low LoD of 1 cell/1 mL. The amine groups in PANI NF help to bind antibody after treating with bovine serum albumin (BSA) to block any nonspecific treatments [103]. 

Guanine and deoxy-guanosine, along with its oxidation products, are important biomarkers for oxidative DNA damage, which is the cause of cancer, degenerative disease, etc. A Pt/GO NF with enhanced surface area was designed through layer-by-layer self-assembly onto a GCE for detection of 8-oxoguanine, which demonstrated an LoD of 0.025 × 10^−9^ M [104]. Similarly, stacked graphene nanofiber (SGNF), acting as an anionic dopant for PPy film, at a disposable SPE, resulted in earlier oxidation of guanine, exhibiting an excellent response in comparison with that of a graphite-doped PPy electrode [105]. Carboxyl-group-functionalized stacked graphene nanofiber (SGNF) was incorporated into PPY as an anionic dopant for the detection of guanine oxidation for DNA hybridization reaction on SPE. The modified SPE was sensitive not only to the earlier oxidation of guanine, but also to H_2_O_2_, which is an important biomarker in the body. This NF-based polymer modifier sensing platform is more sensitive than graphite-doped PPY and, hence, can be applied to detect other important biomarkers. Furthermore, these NFs have good conductivity due to the incorporation of a conductive polymer, such as PPY or SGNF, and can serve as an effective electrode material for electrochemical detection. The high surface area of the NFs leads to increased electron transfer, which leads to a more sensitive detection platform for target biomolecules.

### 3.3. Detection of Biomarkers for Cardiovascular Disease

**Figure 6:**
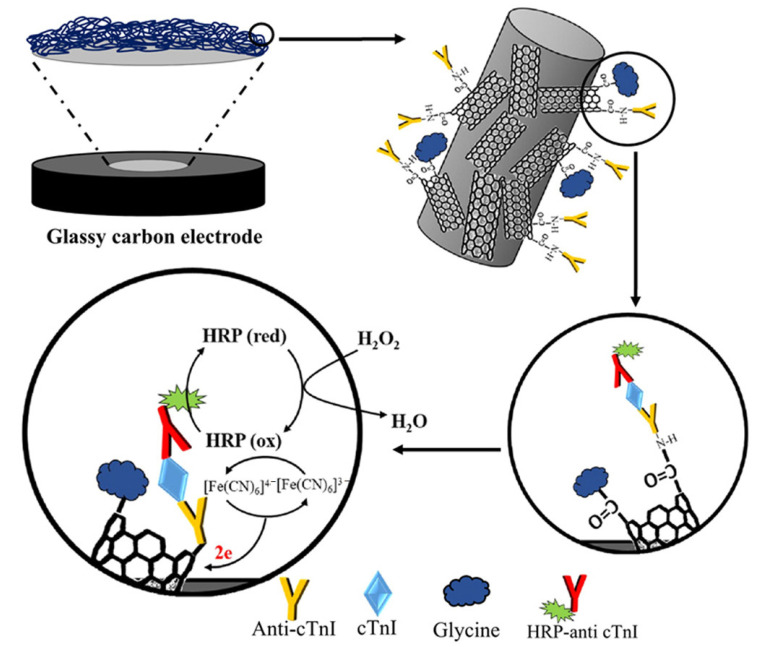
Construction of immunosensor with WNF on GCE and its mechanism of detection. Reprinted with permission from [111]. Copyright (2023) Elsevier B.V.

C-reactive protein (CRP) is an important biomarker used for the early detection of cardiac diseases and is sensitive to systemic inflammation (SI) secreted into the blood stream by liver cells, thus rapidly increasing inflammation by several orders of magnitude. CRP can therefore act as an indicator for timely therapeutic interventions. A biosensor platform was designed by employing vertically aligned carbon nanofibers, where each fiber acted as a nanostructured electrode and formed a 3 × 3 nanoarray. Anti-CRP was immobilized on the NF, which, in the presence of CRP, reduces redox current and charge transfer resistance, establishing itself as a specific and sensitive bioelectrode system. The LoD was found to be 90 pM [106]. Gupta et al. reported a multiplex label-free real-time immunosensor comprising 3 × 3 identical but isolated microelectrode arrays for simultaneous detection of the early marker CRP and postacute myocardial infarction (AMI) markers, such as cardiac troponin–I (cTnI) and myoglobin, by instigating carbodiimide chemistry. The biosensor consists of CNF nanoelectrodes that were vertically grown using plasma enhanced chemical vapor deposition method, which introduced functional groups such as COOH on the surface of the CNFs. The array was immobilized with three specific capture antibodies at the selectively etched surface of the electrode. The electrode showed high sensitivity and selectivity for three selected biomarkers in complex protein mixtures with no false positive signals due to nonspecific protein interactions [107]. Cardiac troponin–I (cTnI), a diagnostic biomarker for myocardial infraction, was detected by immobilizing CNF with anti-cTnI Ab via CV and the EIS method through changes in Ab–Ag interactions. The biosensor could detect cTnI at concentrations as low as ~0.2 ng/mL, which is 25 times more sensitive than enzyme-linked immunosorbent assay (ELISA) [108]. ELISA has low accuracy for the detection of cTn1 and is expensive and laborious. Hence, nanosensors are much needed for filling the gap and rendering reliable results. EIS was employed to detect cTnI by immobilizing molybdenum disulfide/cellulose acetate (CA) NF on SPE. Different concentration of MoS_2_ nanosheets were used and electrospun with CA to fabricate an effective nanobiosensor with CA, providing the porosity that is required for accessing the matrix for its detection. The presence of –OH, –COOH, and –O– groups makes the CA ionic in nature and helps with binding metal NPs to the polymer. The detector could detect 10 fM of cTnI and exhibited 90% stability, even after six weeks [109]. CNTs find wide applications because of increasing the rate of electron transfer and sustaining the biological activity of the bioactive materials on the sensors; however, they exhibit poor dispersibility, which can be avoided when functionalized [110]. NF whiskers embedded onto carboxylated MWCNT (CMWCNT) were developed as a sandwich-type immunosensor on GCE for the detection of cTnI to explore the merits of both NFs and NTs, as shown in Figure 6. The CMWCNTs were immobilized by the antibodies of cTnI and were further conjugated by horseradish peroxidase to form an immunocomplex with cTnI, which was able to detect H_2_O_2_ with an LoD of 0.04 ng mL^−1^. This nanostructured immunosensor showed promise for PoC diagnostics [111]. The functional groups (–OH and –COOH) on CNTs offer electrostatic affinity for analytes. The superior performance of the sensing matrix can be attributed to the synergistic role of CNTs and NFs. They facilitate electron transfer due to their conductive nature and support analyte adsorption. Moreover, the functionalities can develop hydrogen bonds with the analyte and between the surface-adsorbed analytes and their solvent-dissolved species. This results in boosting their regional concentration and enhancing electrocatalytic redox events. 

Poly-ethylenimine (PEI) and glutaraldehyde-modified MWCNTs on SPE promote enhanced electrochemical performance for sensing CRP. This was modified by embedding protein A at the surface of electrode for achieving proper orientation by crosslinking with anti–CRP antibodies by using dimethyl pimelimidate dihydrochloride for the detection of CRP on the surface, forming a sandwich immunosensor [112]. Furthermore, a conductive polymer, PEI, was immobilized on the surface of a Au electrode to covalently bond to carboxylated CNTs for sensing cardiac troponin T(cTnT). EDC/HNC chemistry was used to bind anti–cTnT. This was then immersed in varying concentrations of cTnT in saline solution, which was subsequently incubated in anti-cTnT-HRP tracer. HRP is responsible for the oxidation of H_2_O_2_ produced as a biomarker of cancer, which was detected with a low LoD of 0.033 ng/mL [113]. This was further improved by employing amine-functionalized MWCNTs on SPE for the detection of cTnT conjugated to secondary protein with HRP. The LoD improved to 0.016 ng/mL. The in situ electroactive functionalities, porous texture, and conductive channels at the amine-functionalized MWCNT facilitated the redox reactions via the effective electron/proton transfer of the analyte molecules to its surface in response to the applied potential. Thus, it worked as a highly efficient electrocatalyst to lower the analyte potential and enhance the effective surface area of the sensor. For instance, their oxygen and nitrogen functionalities acted as anchoring sites to form hydrogen bonds, which are responsible for increased analyte adsorption. However, label-free recognition techniques are less time-consuming due to the lack of the need for incubation of the electrode before detection. Hence, research has been focused on developing immunosensor without using labels for antibody–antigen interactions. Hence, amine-functionalized CNT was immobilized as a printing ink on SPE for the detection of cTnT without labelling with secondary proteins by engaging differential pulse voltammetry (DPV), reporting the difference in voltage peak before and after the attachment of cTnT [114]. Similarly, NFs that were synthesized by electrospinning nanostructured camphor sulfonic acid doped with a PANI/polystyrene (PS) blend were developed as a label-free electrochemical sensor functionalized with anti-CRP for detecting CRP on a comb-shelled Au microelectrode chip in a microfluidic device with a low LOD of 100 fg/mL, according to EIS, in a wide linear range of 100 fg/mL^−1^ mg/mL [115]. Nonconductive nanotextured PS was used as a solid support for the immobilization of anti-CRP by van der Waals interaction for sensing CRP in human serum via EIS, measuring the fluctuations in double-layer capacitance. This led to a sensing matrix that was found to be economical and at par with ELISA [116]. 

Mondal et al. developed a biosensor for detecting esterified cholesterol (Ch) by modifying the surface of ITO with electrospun partially aligned anatase TiO_2_ NF mats. Oxygen plasma treatment resulted in the introduction of –COOH and –CHO at the surface of highly porous TiO_2_ NFs, making them hydrophilic. Furthermore, Ch. esterase and Ch.oxidase were introduced via covalent bonds, invoking EDC–NHS chemistry, with an LOD of 0.49 mM and a response time of 20 s, which resulted in enhanced binding of the analyte to the sensing matrix [38]. Similarly, electrospun TiO_2_ NF mats were used as a cell capture immunoassay for the detection of tumor cells in colorectal and gastric cancer patients [57]. The as-synthesized nanofibers have a large surface area to volume ratio, which increases the chance of capturing target molecules. This is important for electrochemical detection, as the signal generated is directly proportional to the number of molecules captured.

### 3.4. Detection of Biomarkers for Infectious Disease 

#### 3.4.1. Detection of Virus via Electrospun NFs 

**Figure 7:**
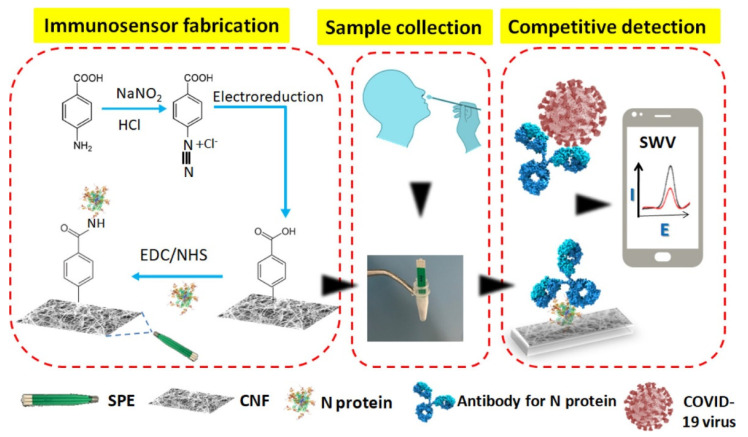
Schematic showing fabrication of cotton-tipped electrochemical immunosensor by functionalizing the CNF with electro-reduced diazonium salt for detection of the viral antigen of COVID-19, and sample collection using the fabricated electrode, detection of target with the help of the competitive assay and SWV technique. Adapted with permission from [124]. Copyright (2023) American Chemical Society.

Middle East respiratory syndrome coronavirus (MERS-CoV) causes severe infection, especially in patients with diabetes and people who are immunocompromised. The disease is spread through direct or indirect contact with Arabian camel and has been found to transmit to humans especially in the Middle Eastern region. Hence, the diagnostics for early detection is crucial. COVID-19 has gained all the attention in the past few years, with the resources for MERS-CoV-2 being directed toward it. MERS-CoV-2 is confirmed via PCR, but most developing countries have limited resources for using it as a diagnostic tool, as it is an expensive instrument that requires highly trained personnel. Furthermore, immunodiagnostic techniques such as ELISA are effective only 7–11 days after the onset of infection, while diagnosis based on antibodies is only feasible in the recovery period. Thus, there is a dire need in the current pandemic situation to develop economical disposable portable nanobiosensors that can minimize the risk of handling the contagious samples and provide a response in a short time. Disposable materials such as paper, textiles, and plastic have been used as flexible materials for fabricating light-weight and low-cost biosensors as PoCs [117,118,119]. These PoCs need to fulfil the ASSURED criteria as promulgated by WHO [120]. Similarly, cotton thread, which allows the flow of liquid via capillary forces, has been developed with the need to construct separate microchannels and external pumps [121,122]. Shimaa et al. reported a PoC testing device produced by using disposable-cotton-modified electrode for the detection of MERS-CoV-2. Carbon SPE (CSPE) was modified with CNF, which was then further functionalized with carboxyphenyl groups by activation with EDC–NHS chemistry for immobilizing the MERS-CoV spike proteins [123]. The simple CNF modification of CSPE leads to enhanced voltammetric signals acquired through a potentiostat on a smart phone. Further functionalization of the CNFs with carboxyphenyl groups leads to the shielding of aryl groups and the development of negative charge, resulting in a decrease in the peak current due to repelling the negatively charged anionic ferro/ferricyanide redox couple. However, when the spiked protein is covalently attached, it again results in an increase in current, as some of the negative charge is neutralized. The modified electrode’s tip is then spun with cotton fiber for swabbing and detecting the spiked protein. This results in the absorption of the body fluid that contains virus into the cotton swab, which, by capillary action after immersion in antibody solution, flows toward the modified biosensor monitored by SWV on a smart phone connected to a portable potentiostat. The competition with free spiked protein and immobilized protein with the virus in the sample results in either a low or high biosensor response, depending on binding events between fixed amounts of virus spike protein and antibody. A high virus content leads to a low current response of the biosensor and vice versa. A miniaturized portable biosensor was thus designed to detect MERS-CoV with a LoD of 0.07 pg/mL^−1^, which was insensitive to proteins from HCoV and influenza, depicting high selectivity. Hence, these electrospun NFs can be functionalized with specific chemical groups that can selectively bind to the target analyte. This selective binding improves the detection specificity, which is crucial in diagnosing a virus. Similarly, the detection of COVID-19 with a PoC device that is fast and easy as well as affordable is one of aims that scientists are attempting to achieve. As the main method of detection of COVID-19, nasopharyngeal samples are collected on swabs to extract RNA by transferring virus into a solution for reverse-transcription PCR. A paper [124] reports nanofiber-functionalized SPE electrografted with diazonium ions and coating the resulting electrode with a cotton padding, which enables the swabbing and detection of a sample from a single matrix in one step by SWV, in the same fashion as in [123], as shown in Figure 7. The LOD was reported as 0.8 pg/mL, demonstrating the successful detection of virus antigen with a modified SPE platform. 

The DNA hybridization method for electrochemical detection by using DNA biosensors is a simple, ultra-sensitive, and easy to fabricate method and is hence the method of choice. Doping enhances the properties of the biosensors [53,125]. Metal oxides with a low bandgap have FET–like behavior, which can be tuned by actuation to improve catalytic properties, resulting in reduced overpotential as well as improved electron transduction at the electrode electrolyte interface [126]. Dengue fever is caused by a mosquito of the Flaviviridae family when it injects the Dengue virus (DENV) into the blood stream, resulting in symptoms ranging from illness, fever, and serious hemorrhagic fever to Dengue shock syndrome, which may lead to mortality and morbidity [127]. Its identification and detection have become all the more important for the prevention of mortality and epidemic outbreaks, especially in developing countries. Reverse-transcription PCR was reported to detect the RNA of Dengue virus [128]; however, it is an expensive technique. Hybridization biosensors for the detection of DENV have been reported [129,130,131], which are more economical and offer on-site rapid response. Polymethyl methacrylate-co-methacrylic acid coated onto electrospun polyhydroxybutyrate (PHB) fibers was employed as a unique biosensing platform for the detection of DENV by employing a modified platform for ELISA. This was compared with the conventional ELISA technique, which is used in clinical practices, despite major shortcomings including irreproducible results and large detection ranges for achieving accurate results. Electrospun fibers provide a large specific area for interactions with biomolecules, while coating with polymers adds a functional group such as –COOH for effective binding events with the antibodies to Dengue virus. This is followed by the physical adsorption of the Dengue antibodies and chemical bonding by utilizing carbodiimide chemistry for the stable conjugation of the antibody. The optimum concentration and proper distribution of the functional groups at the surface of the sensing matrix dictate the effective immobilization of the proteins for the detection of DENV. However, caution is required when introducing functional groups, as too few or too many surface functionalities may lead to the deactivation of the proteins. This technique can be extended to paper-based biodiagnostic POC with high-performance and cost-effective sensing platforms [132]. Tripathy et al. [44] reported electrospun manganese oxide Mn_2_O_3_ nanofibers by using a mixture of PAN/DMF as a sacrificial polymer matrix on GCE for the label–free electrochemical detection of DENV in the zeptomolar range. The modified GCE was introduced with functional groups by immersion in MPA solution for 15 h, which formed a self-assembled layer on the surface of the modified electrode. Further treatment of EDC/NHS was accomplished for the activation of the –COO group, which allowed the immobilization of ssDNA at pH = 8 in a Tris-EDTA (TE) buffer for amide linkage, to establish between the nucleotide amine group and activated –COOH group. The electrode was then probed for the detection of DENV by immersion in a t–DNA complementary sequence for the hybridization reaction to ensue. The biosensor was able to detect Dengue consensus primer with an LoD of 120 × 10^−21^ M. This ultra-sensitivity and facile bioelectrode indicated an outstanding result due to using a low bandgap electrospun manganese oxide nanomaterial. This method can be employed for the detection of any sequence primer by DNA hybridization simply by adapting the matrix with the proper protocol for functionalization. Similarly, Arshad et al. fabricated molecular imprinted polymer (MIP) on a PSNF-functionalized electrospun SPE-based voltammetric sensor for the detection of DENV. This matrix was further modified with dopamine by polymerization, keeping the NS1 protein (specific and sensitive biomarker for DENV infection), with a detection limit as low as 0.3 ng/mL. MIP has specific geometric and chemical fitting cavities that enhance electrochemical transduction [133].

Homogeneously dispersed and hierarchical-flower-like Au microspheres were produced via three-step electrodeposition on GCE modified with self-doped PANI nanofiber for the detection of the cauliflower mosaic virus 35S gene. The modified electrode presented good biocompatibility and a large surface area, with outstanding electron transduction. The DNA biosensor fabricated for the detection of the virus gene via the EIS method by employing DNA hybridization demonstrated an LoD of 1.9 × 10^−14^ M. This DNA biosensor holds promise for the detection of other biomolecules of significance [134]. 

Oxygen-plasma-treated nitrocellulose fibers were employed as a capture membrane for immobilizing antibodies for the detection of viral and bacterial pathogens. This modified surface was designed on a Ag electrode obtained by the spray-deposition method. The electrode was able to detect *E. coli* O157:H7 and bovine viral diarrhea virus by employing confocal laser scanning microscopy and scanning electronic microscopy. This bioelectrode demonstrated excellent detection capability due to its capillary action and efficient functionalization with antibody and can be used for different applications for immune detection [135]. 

The liver is a significant organ that performs different functions in the body. Hepatitis B virus (HBV) causes viral hepatitis, which affects the liver and, due to the mutation of genes, becomes resistant to antiviral drugs [136]. DNA analysis has been carried out by employing surface plasmon resonance, surface-enhanced Raman scattering, and fluorescence techniques and electrochemical methods for the simple and inexpensive detection of HBV [137,138,139]. Electrochemical methods are more economical, sensitive, and simple to use when using Pt–, Au–, and C–paste-based electrodes. Hence, different groups have used nanogold rods on Au electrodes and SWCNTs on Au NPs for the measurement of the DNA sequences of the virus with a low LOD [140]. Niri et al. reported a DNA biosensor based on electrospun CNFs extracted from PAN modified with glutamic acid for the detection of HBV via DNA hybridization. This modified platform was conjugated to probe DNA for HBV. Hence, in a solution of target DNA, the sensor detected HBV in a linear range of 1 × 10^−12^–1 × 10^−6^ M with an LOD of 1.58 × 10^−12^ M [141]. The electrospun CNF makes the biosensor economical, effective, and easier to upscale. 

#### 3.4.2. Detection of Immunoglobulin via Electrospun NFs 

Core–shell Au@Ag nanorods were reported as being biocompatible and having a large surface area for the detection of immunoglobulin IgG, which is the main type of antibody that safeguards the body from infections. Au@AgNRs were readily used as tracing tags by labeling them with anti–HIgG, which forms an immune complex when bound with IgG (Ab1). The nanocomposite of CNFs and NH_2_-terminated polyamidoamine (PAMAM) dendrimer, with an abundance of functional groups, showed excellent binding efficiency with antibodies dispersed onto a GCE. This was incubated with the captured antibodies. The sandwich-type immunosensor resulted in peak current magnification compared with the Ag NPs in bimetallic rods [142] and hence enhanced the sensitivity of the biosensor. The electrospun NFs were found to increase the surface area of the sensing matrix. It was found that NFs are of special interest as they can be functionalized with specific receptors or antibodies that can selectively capture target biomolecules. This makes them highly specific and sensitive for the detection of particular biomarkers.

Table 1 shows the figures of merit for different modified electrode surfaces. It shows that most of the developed electrochemical sensors show very low LODs to detect minute concentrations of the biomarkers in a reasonable linear range. However still research needs to be more focused on improving the stability issues of the developed sensing platforms. 

Glassy carbon electrode (GCE); poly(methyl methacrylate) (PMMA); poly(diallyldimethylammonium chloride) (PDDA); nitrogen-doped carbon nanospheres@carbon nanofibers (NCNS@CNFs); nanofibrous electrode (NFE); epidermal growth factor receptor 2 (ErbB2); α-fetoprotein (AFP); anti-aflatoxin B1 (AFB1); human epidermal growth factor receptor 2 (HER2); laser-scribed graphene (LSG); breast cancer 1 (BRCA1) gene; ribbon conductive nanofibers (RCNFs); human melanoma cell line (SKMEL-2); anti-MC1R Ab-functionalized PANI-NFs-modified screen-printed electrode (MC1R-Ab-PANI/SPE); 8-hydroxy-2’-deoxyguanosine (8-OHdG); C-reactive protein (CRP); vertically aligned carbon nanofibers (VACNFs); cardiac troponin-I (cTnI); carboxylated multiwalled carbon nanotube (CMWCNT)–embedded whiskered nanofibers(WNFs); polyethylene glycol, (PEG); polyethyleneimine (PEI); Middle East respiratory syndrome coronavirus (MERS-CoV); severe acute respiratory syndrome coronavirus 2 (SARS-CoV-2); molecular imprinted polymer (MIP); glutamic acid (Glu); hepatitis B virus (HBV); nonstructural protein 1 (NS1); histidine-rich protein 2 (HRP2); Plasmodium falciparum histidine rich protein-2 (PfHRP-2); glass microballoons (GMBs); cauliflower mosaic virus 35S gene (CaMV35S gene); self-doped polyaniline nanofibers (nanoSPAN)

## 4. Conclusions and Future Perspectives

The use of biosensors in point-of-care (PoC) testing devices has attracted considerable attention in the past few years, mainly because of their high specificity, portability, and relatively low cost. Nanotechnology has given the required push in the field of bio-testing devices, revolutionizing it by introducing smarter, faster, and more reliable sensing platforms for the detection of biomolecules. Moreover, coupling these devices with miniaturized electrochemical transducers has shown great potential for simple, rapid, and cost-effective analysis that can be performed in the field, especially for healthcare, environmental monitoring, and food-quality control. In this arena, NF-based biosensors have shown to boost the sensitivity and specificity of the sensing matrix. Thus, NF-based electrochemical biosensors have shown great potential in the field of PoCs and on-site detection due to their high sensitivity, selectivity, and low cost. Some of the current emerging trends in this field include:

1. Integration of NF-based biosensors with microfluidic devices to create integrated systems that can perform rapid, sensitive, and multiplexed analyses. This trend is driven by the need for the miniaturization, portability, and automation of biosensors.
2. Label-free detection of biomarkers is an emerging trend in the field of PoC and on-site electrochemical detection. Label-free biosensors can detect biomarkers without the need for labeling agents, which can simplify the detection process and reduce the cost of biosensors. Researchers are exploring the use of NF-based biosensors for label-free detection of biomarkers.
3. The integration of nanofiber-based biosensors with wearable devices such as smart watches, patches, and clothing allows the continuous monitoring of biomarkers and the wireless transmission of data to healthcare professionals for real-time monitoring and diagnosis.
4. Multiplexed detection of biomarkers enables the simultaneous detection of multiple biomarkers, which can provide a more comprehensive picture of a patient’s health status. Researchers are exploring the use of nanofiber-based biosensors for the multiplexed detection of biomarkers.
5. Three-dimensional printing technology is being employed to fabricate NF-based biosensors with complex geometries and structures. This allows for the creation of highly specific and selective biosensors that can simultaneously detect multiple targets.
6. The integration of NF-based biosensors with smartphones and other portable devices can provide real-time monitoring and analysis of biomarkers, which can improve the diagnosis and treatment of diseases.
7. The use of artificial intelligence and machine learning algorithms is becoming increasingly popular for the analysis of the large datasets generated by NF-based biosensors. These algorithms can identify patterns and correlations in the data, enabling the development of highly accurate and reliable diagnostic tools.

Furthermore, electrospun NFs provide excellent support for enzyme immobilization, providing a large surface areas and porosity. The functionalization of the surfaces for attachment or entrapment, providing favorable environments around the biomolecules to improve enzyme stability and activity, has been successful. The design and fabrication of NFs-based biosensors is an expanding area of research but is still in its infancy. At present, most of the biosensors use enzymes as sensing elements for electrochemical transduction, while nonenzymatic platforms are required for finding a solution to the tedious immobilization of enzymes and their ultimate denaturation. This is an active research area, and overcoming these challenges will be crucial in advancing their practical applications.

## 5. Challenges Facing NF Based Electrochemical Sensors for Application in Medical Diagnostics

Although great improvement has been achieved in the field of electrochemical biosensing, there are still some challenges to overcome, especially concerning the improvement in sensing materials and miniaturization. The versatility of the recognition system, achieved by improving the transduction process, is considered a leap in the field of biodetection, which is fast moving toward one-cell detection. It is projected that NFs will occupy an important role in future applications for biomarkers of medical importance, being economical and having the ability to be scaled up. Electrospinning has been the most commonly used technique to upscale the production of NFs and offers a wide scope for applications in different fields. Furthermore, it allows the control of the morphology of the NFs by changing the properties of the electrospinning parameters, which helps to develop functional NFs most suited for various applications. However, still there are some challenges facing the production of NFs that must be overcome.

The fabrication of nanofibers is a complex and time-consuming process that requires specialized equipment and expertise. The fabrication of highly functional and high-quality NFs for commercial purposes may take some time, given the limitations regarding its reproducibility and the scalability of the fabrication process. The biocompatibility of the synthesized NFs must be considered in relation to the environment. In this regard, green materials need to be emphasized; hence, using ecofriendly polymers in the electrospinning process can help reduce the environmental impact of polymer production and disposal, as these polymers are often biodegradable and/or derived from renewable resources. Furthermore, the needleless solvent-free electrospinning technique is promising, as it reduces or eliminates the need for solvents, which can be harmful to the environment and human health, thereby making the technology sustainable. Here, ionic liquids, which are nonvolatile and nonflammable, unlike conventional solvents, can be used for electrospun polymers. Similarly, supercritical carbon dioxide can be employed as the spinning medium instead of liquid solvents, which is a nontoxic, nonflammable, and inexpensive alternative to traditional solvents. However, the use of this technology is still in its proof-of-concept stage, and more rigorous study is needed to improve medical diagnostics. NFs with a porous structure can allow the faster diffusion of analytes to the sensing element, leading to a more rapid response time. Moreover, the alignment and orientation of the nanofibers can affect the sensitivity and selectivity of the sensor. Aligned NFs can provide directional sensitivity, while randomly oriented nanofibers can provide more uniform sensitivity. The morphology of nanofibers plays a critical role in the performance of electrochemical sensors. By optimizing the nanofiber morphology, it is possible to enhance the sensitivity, selectivity, and response time of the sensor, which is an area of immense interest. Furthermore, NF–based electrochemical biosensors are often sensitive to changes in the surrounding environment, including temperature, humidity, and pH, which can degrade the sensing matrix. These factors can affect the stability and performance of the sensor over time. Achieving real-time monitoring with NF-based electrochemical biosensors can be challenging due to the slow response time of some sensors in real samples, which is the foundation on which PoCs have been developed. This may be attributed to the presence of interfering species that can affect the selectivity of the sensor and lead to false-positive or false-negative results. Hence, active research is required to overcome these challenges. Thus, NF–based biosensors need to be highly selective to the target biomarker of interest and require more rigorous research. Developing a biosensor involves technical difficulties, making the process expensive and cumbersome, as sensors with different antigens/antibodies need to be considered; however, nanotechnology has given it the boost required. Soon, single nanobiosensor will be able to diagnose and detect multiple analytes/ions in one single assay.

## Figures and Tables

**Table 1:** Different parameters of modified electrochemical sensors for detection of different biomarkers.

Sr #	Disease	Analyte	Method	Electrode	Linear Range	LOD	Recovery	Stability	Ref.
1.	Diabetic	Glucose	Amperometry	ZnO/PVP NF/Au	0.25–19 mM	1 µM	-	120 days	[37]
2.	Diabetic	Glucose	Amperometry	Mn_2_O_3_-Ag NF/GCE	up to 1.1 mM	1.73 µM	-	-	[42]
3.	Diabetic	Glucose	Amperometry	PMMA–MWCNT (PDDA)/GOx–NFE	20 µM–15 mM	1 µM	98%	35 days	[72]
4.	Diabetic	Glucose	CV	NCNS@CNFs	12–1000 μM	2 µM	-	-	[80]
5.	Diabetic	Glucose	Amperometry	CuCo-CFs	0.02–11 mM	1.0 µM	95%	30 days	[61]
6.	Diabetic	Glucose	Amperometry	CuO/PCL@PPy/ITO	0.002–6 mM	0.8 μM	96.36%	25-days	[63]
7.	Cancer	ErbB2	EIS	ZnO NF	1.0 fM–0.5 µM	1 fM	95%	50 days	[55]
8.	Cancer	AFP	CV	IrO_x_ NF/chitosan	0.05–150 ng/mL	20 pg/ ml	-	15 days	[58]
9.	Hepatocellular carcinoma	AFB1	EIS	BSA/anti-AFB1/ µchannel/C-Pt	1 pg/mL–10 µg/mL	1 pg/ml	-	40 days	[70]
10.	Cancer	HER-2	SWV	LSG-AuNS	0.1–200 ng/mL	0.008 ng/mL	107%	-	[88]
11.	Cancer	BRCA1	EIS	RCNFs-MWCNTs/CPE	5 pM–14 nM	2.4 pM	105%	14 days	[92]
12.	Cancer	H_2_O_2_	Amperometry	Au-Ag/Co_3_O_4_ NFs	0.05–5000 µM	0.01 µM	-	30 days	[93]
13.	Cancer	SKMEL-2	DPV	MC1R-Ab-PANI/SPE	15–7000 cells/5 mL	1 cell/1 mL	-	9 days	[103]
14.	Cancer	8-OHdG	DPV	Pt-NFs/GO/GCE	0.0007–2.00 µM	0.025 nM	101%	7 days	[104]
15.	Cardiac	CRP	EIS	VACNFs	up to 42 nM	90 pM	-	-	[106]
16.	Cardiac	Cholesterol	CV	cTiO_2_–NF/ITO	25–400 mg/dL	0.49 mM	97%	120 days	[38]
17.	Myocardial infarction	cTnI	EIS	CNFs	0.25–1.0 ng/mL	0.2 ng/mL	-	-	[108]
18.	Myocardial infarction	cTnI	CV	PS/CMWCNTs/PEG NFs/GCE	0.5–100 ng/mL	0.04 ng/mL	-	10 days	[111]
19.	Myocardial infarction	cTnI	Amperometry	*f*CNT/PEI/Au	0.1–10 ng/mL	0.033 ng/mL	95%	-	[113]
20.	Myocardial infarction	cTnI	DPV	CNT-SPE	0.0025–0.5 ng/mL	0.0035 ng/mL	95%	-	[114]
21.	COVID	MERS-CoV	SWV	CNFs/SPE	0.1 pg/mL–1 μg/mL	0.07 pg/mL	96%	7 days	[123]
22.	COVID	SARS-CoV-2	SWV	CNFs/SPE	1–1000 ng/mL	0.8 pg/mL	95%	-	[124]
23.	Hepatitis	HBV	CV	Glu-CNFs	1 pM–1 µM	1.58 pM	-	15 days	[141]
24.	Dengue	NS1	EIS	MIP/SPE	1–200 ng/mL	0.3 ng/mL	97%	-	[133]
25.	Dengue	DNA	DPV	Mn_2_O_3_ NF	1 aM–1 µM	120 zM	90.5%	23 days	[44]
26.	Malaria	HRP2	EIS	*f*Cu-ZnO NFs/GCE	10 ag/mL–10 µg/mL	0.6 ag/ml	96%	60 days	[53]
27.	Malaria	*Pf*HRP2	Colorimetry	CNF/GMB	0.01–10 ng/mL	0.01 ng/mL	-	-	[54]
28.	Mosaic	CaMV35S	EIS	Au/nanoSPAN/GCE	10 pM–1 µM	19 fM	-	-	[134]

## Data Availability

No new data were created or analyzed in this study. Data sharing is not applicable to this article.
